# RAD-QTL Mapping Reveals Both Genome-Level Parallelism and Different Genetic Architecture Underlying the Evolution of Body Shape in Lake Whitefish (*Coregonus clupeaformis*) Species Pairs

**DOI:** 10.1534/g3.115.019067

**Published:** 2015-05-21

**Authors:** Martin Laporte, Sean M. Rogers, Anne-Marie Dion-Côté, Eric Normandeau, Pierre-Alexandre Gagnaire, Anne C. Dalziel, Jobran Chebib, Louis Bernatchez

**Affiliations:** * Institut de Biologie Intégrative et des Systèmes (IBIS), Département de Biologie, Université Laval, Pavillon Charles-Eugène-Marchand, Québec, G1V 0A6, Canada; † Department of Biological Sciences, University of Calgary, 2500 University Drive N.W., Calgary, Alberta, T2N 1N4, Canada; ‡ Institut des Sciences de l’Evolution – Montpellier (ISEM - CNRS UMR5554), SMEL, 2 rue des Chantiers, 34200 Sète, France

**Keywords:** adaptive radiation, parallel evolution, fish body shape, geometric morphometrics, genotyping-by-sequencing

## Abstract

Parallel changes in body shape may evolve in response to similar environmental conditions, but whether such parallel phenotypic changes share a common genetic basis is still debated. The goal of this study was to assess whether parallel phenotypic changes could be explained by genetic parallelism, multiple genetic routes, or both. We first provide evidence for parallelism in fish shape by using geometric morphometrics among 300 fish representing five species pairs of Lake Whitefish. Using a genetic map comprising 3438 restriction site−associated DNA sequencing single-nucleotide polymorphisms, we then identified quantitative trait loci underlying body shape traits in a backcross family reared in the laboratory. A total of 138 body shape quantitative trait loci were identified in this cross, thus revealing a highly polygenic architecture of body shape in Lake Whitefish. Third, we tested for evidence of genetic parallelism among independent wild populations using both a single-locus method (outlier analysis) and a polygenic approach (analysis of covariation among markers). The single-locus approach provided limited evidence for genetic parallelism. However, the polygenic analysis revealed genetic parallelism for three of the five lakes, which differed from the two other lakes. These results provide evidence for both genetic parallelism and multiple genetic routes underlying parallel phenotypic evolution in fish shape among populations occupying similar ecological niches.

Understanding the genetic basis of adaptation of complex phenotypic traits is a major goal in ecologic and evolutionary genetics. In particular, the mechanisms linking genotype to phenotype can be complex (*e.g.*, pleiotropy, polygeny, epistasis) and result in genetic architectures that may either constrain or promote the effects of selection on beneficial alleles in a new environment (Gompel and Prud’homme 2009; Elmer and Meyer 2011; Losos 2011; Conte *et al.* 2012; Rogers *et al.* 2013). Therefore, elucidating whether parallel adaptation to similar environments relies on similar genetic changes is crucial to better understand how populations can adapt to different environments.

Parallel evolution refers to the evolution of ecologically and phenotypically similar traits in independently derived populations or species (Endler 1986; Losos 2011; Conte *et al.* 2012; Merilä 2014). Parallel evolution has best been documented in microbial experimental evolution studies (Rainey and Travisano 1998; Gerstein *et al.* 2012; Heron and Doebli 2013). However, it is a relatively common feature observed in fishes inhabiting lakes in north temperate regions (Mcphail 1984; Bernatchez and Dodson 1990; Skùlason and Smith 1995; Taylor 1999; Østbye *et al.* 2006; Laporte *et al.* 2011; Elmer *et al.* 2014). Evidence for phenotypic parallelism is commonly used to infer the possible role of adaptive mechanisms being involved, because the probability that such phenotypic transitions occurred multiple times only through stochastic processes (*e.g.*, genetic drift) typically is considered to be low (Schluter 2000; Nosil 2012).

Body shape is a complex phenotypic trait composed of a suite of underlying characters that often are genetically based but also may vary by direct environmental induction (Proulx and Magnan 2004; Albert *et al.* 2008; Klingenberg 2010; Rogers *et al.* 2012; Rogers and Jamniczky 2014). In fishes, shape differentiation can influence a variety of performance traits such as prolonged or burst swimming, feeding efficiency, and mating displays (Webb 1982, 1984; Langerhans and Reznick 2010; Walker 2010). Moreover, the strength of selection acting on whole-animal performance may vary among habitats. For examples, a slender, fusiform body shape reduces drag and may be selected in fish that swim long distances, a stout caudal peduncle allows for faster, more powerful burst swimming that can be beneficial for fish that ambush their prey or those that sprint to escape predators, and larger eyes and a terminal mouth increase feeding efficiency in fish that feed on small prey and may evolve via natural selection in planktivorous fish (Webb 1982, 1984; Langerhans and Reznick 2010; Walker 2010; Willacker *et al.* 2010). Identifying the genetic mechanisms by which fish shape evolves would contribute to improve our understanding of adaptation for a complex, ecologically important phenotypic trait.

The Lake Whitefish (*Coregonus clupeaformis*) species complex exhibits adaptive divergence in association with shape contributing to species-pairs referred to as “normal” and “dwarf.” The “normal” species occupies a benthic niche whereas the derived “dwarf” species is specialized for a limnetic niche (Bernatchez and Dodson 1990; Lu and Bernatchez 1999; Landry *et al.* 2007; Landry and Bernatchez 2010). The dwarf species has evolved independently and repeatedly from the normal ones in several lakes in the St. John River Drainage (Québec, Canada, and Maine, USA) after the last glacial maximum [∼12,000 years ago or about 3000 generations (Bernatchez and Dodson 1990; Pigeon *et al.* 1997; Lu *et al.* 2001; Bernatchez *et al.* 2010)]. The species differ in adult body size and several ecologic and physiologic traits associated with occupying distinct trophic niches (Bernatchez 2004; Bernatchez *et al.* 2010; Evans and Bernatchez 2012; Evans *et al.* 2012; Evans *et al.* 2013). Indeed, these differences match ecologic predictions for habitat use (Willacker *et al.* 2010; Harrod *et al.* 2010; Ozerov *et al.* 2015). Shape differentiation resulting in specialization to benthic and limnetic niches is expected to have evolved in these species pairs, but whether similar genetic changes underlie the evolution of body shape in different replicate lakes is unknown.

The first objective of this study was to test for shape differentiation and parallelism at the phenotypic level between sympatric Whitefish species that evolved independently in five geographically isolated lakes using geometric morphometrics. The second objective was to identify quantitative trait loci (QTL) underlying these same shape traits in a backcross family. We then examined the putative functions of genes associated with QTL toward proposing elucidating the potential molecular mechanisms underlying fish shape differentiation. As third objective we tested for evidence of genetic parallelism among independent wild populations by using both a single-locus method (outlier analysis) and a polygenic approach (analysis of covariation among markers) at shape QTL.

## Materials and Methods

### Sampling, experimental crosses, and genetic mapping

A total of 300 wild Whitefish were sampled from five lakes in the Saint John river basin harboring sympatric Whitefish species pairs using gill nets between June and July 2010 (Table 1). Between 50 and 67 specimens were collected from each lake (Table 1). These were used to determine whether body shape differentiation was present between dwarf and normal Whitefish of a given species pairs and if parallelism at the phenotypic level was present among lakes.

**Table 1:** Geographic sampling coordinates, dates, and sample sizes of dwarf and normal Whitefish photographed in the five lakes surveyed in this study

Lakes	Geographic Coordinates	Date	Normal	Dwarf	Total
Cliff	46°24´20 N	June 2010	25	35	60
	69°15´60 W				
East	47°10´42 N	July 2010	35	33	68
	69°32´52 W				
Indian	46°15’24 N	June 2010	24	31	55
	69°16´49 W				
Témiscouasta	47°39´58 N	July 2010	34	33	67
	68°49´22 W				
Webster	46° 09´18 N	June 2010	25	25	50
	69° 05´17 W				
Total	−	−	143	157	300

A laboratory-raised backcross (BC) family derived from the *F1 hybrid* x *dwarf* cross previously used for QTL mapping (Rogers and Bernatchez 2007) was used to map shape QTL in this study. A total of 198 progeny were produced, 102 of which were subsequently available for constructing a second generation linkage map containing 3438 single-nucleotide polymorphisms (SNP) genotyped by restriction site−associated DNA (RAD) sequencing and assigned to 40 different linkage groups (LGs) with an average resolution of 0.89 cM between mapped markers (mapping details provided in Gagnaire *et al.* 2013a).

### Measuring fish shape and landmark positioning

**Figure 1:**
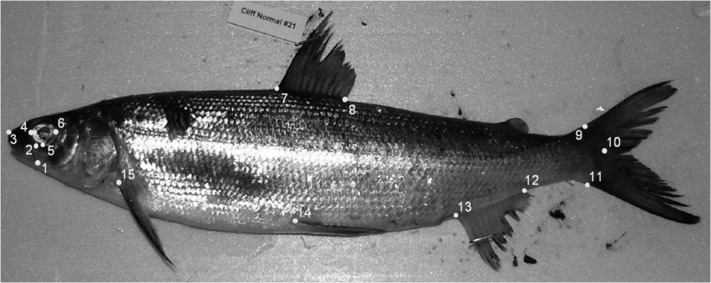
Position of 15 landmarks (30 x, y coordinates) used to study body shape in Lake Whitefish (1: lower extent of distal maxilla; 2: upper extent of distal maxilla; 3: tip of snout; 4: anterior extant of orbital; 5: ventral extant of orbital; 6: anterior extant of orbital; 7: anterior insertion of dorsal fin; 8: posterior insertion of dorsal fin; 9: dorsal insertion of caudal fin; 10: base of caudal fin; 11: ventral insertion of caudal fin; 12: posterior insertion of anal fin; 13: anterior insertion of anal fin; 14: anterior insertion of pelvic fin; 15: anterior insertion of pectoral fin).

Immediately after euthanization, digital photographs of the left side of the 300 wild and 102 BC fish were taken with a Nikon Coolpix P7700 camera to avoid shape deformation that can be associated with preservation. Fish were placed on a Styrofoam board with fins extended and fixed with needles. Fifteen landmarks were digitized on each image using tpsDig v2.16 (Rohlf 2010) to quantify shape differences between individuals (Figure 1). Landmarks were chosen for an optimal coverage of the body (Zelditch *et al.* 2004), reflecting characteristics expected to be under differential selection between limnetic and benthic species (Webb 1982, 1984; Willacker *et al.* 2010).To preserve information on shape differences among fish and to remove information unrelated to shape (*i.e.*, scale, position, and orientation), a partial generalized procrustes analysis superimposition was first applied (Rohlf and Slice 1990; Dryden and Mardia 1998). This resulted in 15 × (abscissa) and 15 y (ordinate) coordinates providing 30 phenotypic traits for further analyses. All protocols were in accordance with the Canadian Council for Animal Care.

### Testing for fish shape differentiation and parallelism in the wild

We first estimated fish shape from the superimposed coordinates projected on a k × p − 4 dimensional principal component analysis (PCA) (k = number of coordinates; *P* = number of landmarks; the four dimensions being lost during the Procrustes superimposition; see Claude 2008) on the 300 wild Whitefish. We used a broken-stick distribution to determine the number of informative principal components (PCs) (Legendre and Legendre 1998). Euclidian distance and angles between the five lines connecting species pairs and the first axis of a between-group PCA were computed using the *groupPCA* function in R/Morph (Schlager 2014). Euclidian distance and angle comparison serve respectively to quantify the fish shape differentiation and the extent of parallelism among species pairs. To visualize the effects of species identity on fish shape for each lake, the juxtaposition of the mean shape of the fish between species from the same lake was conducted.

In addition, a multivariate analysis of variance (MANOVA), from PC scores of the informative axes, was then run to test for the effect of “species” (Dwarf or Normal), “lake,” “sex” (male or female), and their interactions on fish shape. Identification of sex was performed by visual inspection of the gonads and/or by a sex determination locus for Whitefish (locus 82008; Gagnaire *et al.* 2013a) in cases in which the sex was not determined anatomically. To test for significant shape differentiation among species pairs, we ran five independent MANOVAs for each lake with the explanatory variables “species,” “sex,” and their interaction. Shape differentiation was also assessed by verifying the significance of the Euclidian distance with 10,000 permutations using the *groupPCA* function in R/Morph (Schlager 2014).

### The effect of sex on fish shape in laboratory-raised conditions

Identification of sex for BC fish also was performed by visual inspection of the gonads (Rogers and Bernatchez 2007). A MANOVA was then run on fish shape (response variable) with the explained variable sex to test for an effect of sex. Differences in fish shape were visualized with the juxtaposition of the mean fish shape of sexes. All statistical analyses were performed with R software v2.15.1 (Claude 2008; R Core Team 2014).

### Shape QTL analyses

Data files, including phenotypes and genotypes of laboratory-raised Whitefish, were created with the four-way cross format and imported with the *read.cross* function in R/qtl (allowing for four segregating alleles; Broman *et al.* 2003). Genotype probabilities were estimated with the *calc.geneprob* function and a step size of 1 cM for interval mapping. We used the 30 shape coordinates directly instead of shape principal components for QTL mapping, because although the principal components are orthogonal, they do not correspond to independent dimensions of genetic variation (Albert *et al.* 2008; Rogers *et al.* 2012; Berner 2012). We scanned the genetic map for QTL using the Haley-Knott regression method (Haley and Knott 1992) in R/qtl with the *scanone* function (Broman *et al.* 2003). For each of the 30 shape coordinates, a significance threshold was determined for each LG (at 1% significance level) and genome-wide (at 5% significance level) using 10,000 permutations. Finally, the percent variance explained (PVE) of each QTL was computed with the *fitqtl* function and we used the 1.5 LOD support to delimit the confidence interval of each QTL.

### Annotation of body shape QTL

As QTL were genotyped by RAD sequencing, we were able to map these sequenced regions onto a draft Lake Whitefish genome (Pavey *et al.* unpublished data) to gain insight into the functions of some of the genes associated with shape differentiation QTL. This was done using the *blastn* program with the SNP RAD sequences (63 bp) associated with QTL against the assembled contigs of the draft Lake Whitefish genome. Although this genome is not yet published, its quality is sufficient to ensure reliable annotations of genes in subsequent steps (100× coverage, total scaffolds size = 1.3 Gb, N scaffolds = 464 428, scaffold N50 = 3802 bp). The sequence of scaffolds used in this study is presented in Supporting Information, Table S3. When multiple hits were found, only the contig with the greatest e-value was retained. If multiple hits had equal e-values, all contigs were retained for subsequent steps. We then annotated genome scaffolds using the function *blastx* against the Swiss-Prot database (last accessed March 21, 2015), keeping again only the best hit. If multiple hits with equal e-values were found, these were kept only when the annotations were similar (*e.g.*, same protein in different species). We used this two-step procedure to minimize false positives and multiple hits of our SNPs against public databases due to the short length of RAD sequences.

### Testing for selection on shape QTL

A total of 200 wild Whitefish (20 for each of the 10 species/lake groups) were genotyped in a previous study by RAD sequencing for the 3438 mapped markers from the same 5 lakes sampled in this study. Details are provided in Gagnaire *et al.* (2013b). We used these genetic data to test for selection on shape QTL. To determine whether a shape QTL was potentially under divergent selection (single-locus approach), the extent of genetic differentiation between each of the five sympatric species pairs was compared with neutral predictions obtained using coalescent simulations in Arlequin v3.5.1 software (*i.e.*, five nonhierarchical Fdist analyses; Excoffier *et al.* 2009; Excoffier and Lischer 2010). The test was conducted independently for each lake. Corrections for multiple comparisons were performed by controlling the false discovery rate at 5% (Benjamini and Hochberg 1995) with the function *p.adjust* in R v3.0.3 (R Core Team 2014). The number and identity of outlier QTL or associated SNPs was then compared across lakes to evaluate the extent to which parallelism in fish shape is associated with repeated divergent selection at body shape QTL.

Under quantitative genetic theory, rapid adaptation of complex traits is considered highly polygenic (Pritchard *et al.* 2010; Messer and Petrov 2013). It is expected that simultaneous selection of variants at many loci (*i.e.*, polygenic adaptation) will result in subtle variation in allelic frequencies on several covarying loci, yielding a combined effect greater than the effect of individual loci on the phenotype (McKay and Latta 2002; Pritchard *et al.* 2010; Le Corre and Kremer 2012; Bourret *et al.* 2014; Pavey *et al.* 2015). Such subtle changes in allelic frequency are not expected to be detected by genome scan methods that are based on the classical hitch-hiking model (Maynard Smith and Haigh 1974; Pritchard *et al.* 2010; Messer and Petrov 2013; Kemper *et al.* 2014). Therefore, we also tested for the occurrence of a group of covarying markers (which we refer to as a “polygenic approach”) among the 138 body shape QTL that differentiated the two species among the five lakes (see the section *Results*). To search for a group of covarying markers across population pairs, we used the randomForest function implemented in the Random Forest R package (Liam and Wiener 2002). The Random Forest algorithm is a tree-based ensemble machine learning tool more suited to detect evidence of polygenic adaptation since it search for correlation and interactions among loci (Goldstein *et al.* 2011; Boulesteix *et al.* 2012). The efficiency of Random Forest approach in finding a group of covarying markers that differentiate complex traits has been shown in several medicine and agriculture studies (Shi *et al.* 2005; Cordell 2009; Tang *et al.* 2009; Xu *et al.* 2011; Poland *et al.* 2012; Mokry *et al.* 2013; Jarquín *et al.* 2014) but still infrequent in evolutionary molecular [but see Brieuc *et al.* 2015; Pavey *et al.* 2015)]. As recommended by Strobl *et al.* (2009) and Chen and Ishwaran (2012), a total of 100 forests (runs) sets with different seed numbers were computed to ensure randomness of the test. Each run had a total of 10,000 trees. The “importance of markers” (*i.e.*, an indicator of how a marker in interaction with other markers will successfully classify an individual) was used to select a set of covarying markers which we subsequently refer to as “important markers” (*sensu* Chen and Ishwaran 2012), for comparison with the single-locus approach. We added the 99% confidence interval of the 100 runs to this threshold in the selection of important markers to decrease the probability of type I error. The proportion of trees classifying an individual in the dwarf species group and the classification error rate of the Random Forest analysis were used as criteria to determine the presence of similar genetic changes among population pairs. It is expected that the proportion of such trees (which we subsequently refer to as “proportion of votes”; *sensu* Chen and Ishwaran 2012) will be high for dwarf individuals and low for normal individuals. Classification error rate should also be low if similar genetic changes are present among species pairs.

Further evidence of genetic parallelism was searched by calculating mean *F*_ST_ estimates of the important markers between normal and dwarf species in each lake. It has been documented that dwarf Whitefish evolved postglacially [12,000 years before present (ypb)] from the normal ones after a secondary contact in the St. John River basin (Bernatchez *et al.* 2010). If genes that altered fish shape have differentiated under a recent polygenic adaptation, it will thus be expected to observe low F_ST_ for the important markers. Moreover, if true genetic parallelism occurs among species pairs, we predicted that a Random Forest analysis should correctly assign Whitefish of a given species pair even if these fish are not included in the identification of important loci. Consequently, we i) computed a Random Forest analysis without a given species pair, ii) test the ability of the important markers obtained in the assignment of the excluded species pair individuals using the software GeneClass2 (Piry *et al.* 2004), and iii) redo this procedure for all species pairs. All computations were performed in R v3.0.3 (R Core Team 2014).

## Results

### Fish shape parallelism in the wild

**Figure 2:**
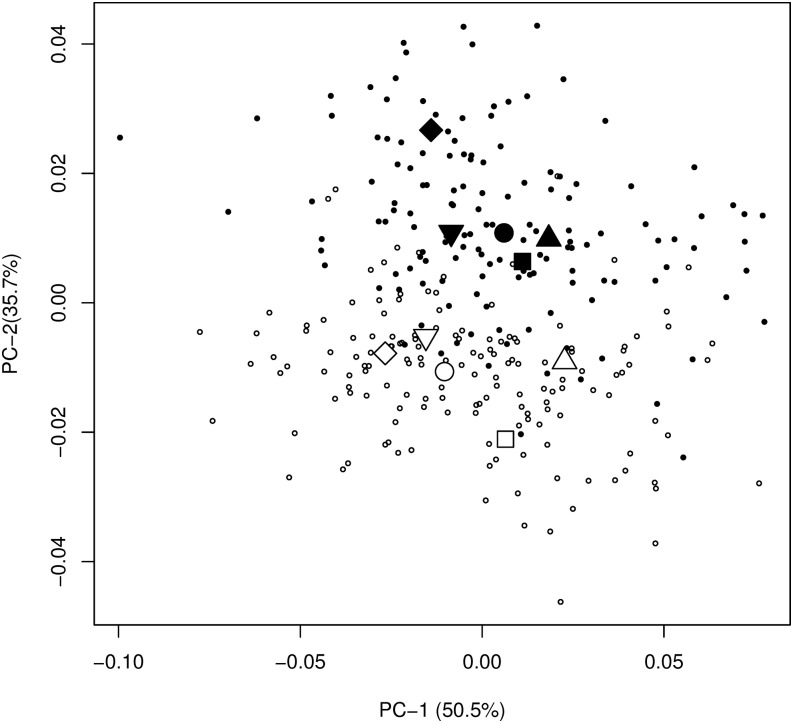
Fish shape variation among individuals along PC1 and PC2 in all five lakes. Each dot represents an individual (black color = normal species; white color = dwarf species). The means fish shape for each species lakes combination were also indicated (circle = Cliff; square = East; diamond = Indian; upper triangle = Témiscouata and lower triangle = Webster).

**Figure 3:**
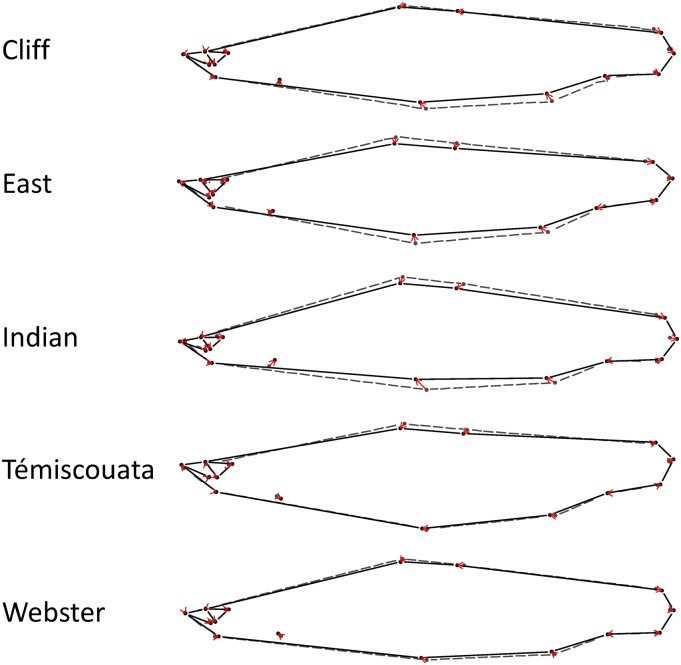
Mean shape comparisons between normal (white dot; dashed line) and dwarf Whitefish (black dot; full line) for Cliff, East, Indian, Témiscouata, and Webster lakes. Red arrows show how shape changes from normal to dwarf Whitefish.

Broken-stick distribution indicated that only the two first PCs were informative. The first axis represents 50.6% of the variation and show variation among lakes (Figure 2). With the exception of Témiscouata Lake, dwarf Whitefish species also showed lower mean score on this axis than normal Whitefish (Figure 2). The second axis represents 35.7% of the variation and showed a clear distinction between all dwarf and normal Whitefish (Figure 2). Euclidian distance showed an increasing gradient in the following order: Webster (0.020; *P*-value = 0.032), Témiscouata (0.021; *P*-value < 0.001), Cliff (0.028; *P*-value < 0.001), East (0.032; *P*-value < 0.001), and Indian (0.038; *P*-value < 0.001). This gradient roughly correspond to the one observed with the juxtaposition of mean shape (Figure 3). In addition, all between-group PCA angles point in a similar direction (9−55°). Interestingly, the lower angle observed (Cliff: 9°) is linked to a ventral thinning in dwarf in comparison to normal Whitefish (Figure 3), the greater angle (Témiscouata: 55°) is linked to a dorsal thinning in dwarf (Figure 3) and the intermediary angles (Indian: 18°, Webster: 22°, and East: 25°) are linked to both ventral and dorsal thinning (Figure 3). Dwarf Whitefish also have bigger eyes and longer tails than the normal fish in all of the five lakes (Figure 3).

The effect of both “species” (dwarf *vs.* normal) and “lake” on fish shape were highly significant (*P* < 0.001) (Table 2). The interaction “species × lake” and “lake × sex” also were significant and a trend of a possible effect of “sex” was observed (*P* < 0.1) (Table 2). As observed with Euclidian distance, phenotypic differentiation between dwarf and normal Whitefish was significant according to the five within-lake MANOVAs (Table 2). Effect of “sex” also was marginally significant in Témiscouata Lake. The effect of “sex” on shape in laboratory-raised fish was also significant (df: 1, 71; Pillai = 0.57; approx F: 3.55; P-value < 0.001). In comparison with males, females have smaller eyes, a deeper body, a shorter tail, and a more anterior insertion of the pectoral fin (Figure S1). Given this and also the fact that previous transcriptomics studies on Whitefish revealed a pronounced sex effect on expression QTL (Derôme *et al.* 2008; Whiteley *et al.* 2008), the identification of shape QTL was performed by considering sex as a cofactor.

**Table 2:** Effect of both “species” (dwarf *vs.* normal) and “lake” on fish shape

Effect of	DF	Approx F	P-value
All lakes*^a^*			
Species	1, 215	161.25	<0.001*
Lake	4, 432	11.97	<0.001*
Sex	1, 215	2.61	0.076**
Species × lake	4, 432	2.75	0.006*
Species × sex	1, 215	0.37	0.691
Lake × sex	4, 432	2.14	0.031*
Species × lake × sex	4, 432	0.99	0.444
Within lake*^b^*			
Cliff			
Species	1, 29	48.10	<0.001*
Sex	1, 29	0.67	0.519
Species × sex	1, 29	0.79	0.465
East			
Species	1, 55	40.13	<0.001*
Sex	1, 55	1.83	0.171
Species × sex	1, 55	0.28	0.756
Indian			
Species	1, 35	90.05	<0.001*
Sex	1, 35	2.26	0.119
Species × sex	1, 35	1.86	0.171
Témiscouata			
Species	1, 48	17.56	<0.001*
Sex	1, 48	3.15	0.051^+^
Species × sex	1, 48	1.13	0.332
Webster			
Species	1, 44	27.41	<0.001*
Sex	1, 44	1.33	0.275
Species × sex	1, 44	1.18	0.317

MANOVA, multivariate analysis of variance.

a Effect of species identity (Dwarf *vs.* Normal), lake, sex, and their interaction on the two first axes of a PCA on 15 landmarks representing fish shape tested with a (MANOVA).

b Five independent MANOVAs (one for each lake) testing for the effect of species identity, sex and their interaction on the same response variables

* Significant result (*P*-value < 0.05).

** Marginally significant result (P-value < 0.1)

### Whitefish shape QTL: laboratory-reared Whitefish

QTL analysis detected 138 QTL that were significant at the LG level, including 26 that also were significant at the genome-wide level (Table S1). There was an average of 3.40 (SD ± 2.57) QTL per LG and 5.67 (SD ± 1.86) QTL per shape coordinate (Table S1). Only three of the 40 LGs did not contain any shape QTL (LG20, 22, and 30) and all shape coordinates had a minimum of 3 QTL (Table S1). The average distance between a QTL and a RAD marker was 0.17 (SD ± 0.43) cM (Table S1).

### Annotation of shape QTL: laboratory-raised Whitefish

Of all 138 SNPs linked to QTL, 113 (81.9%) mapped against the draft assembly of the Lake Whitefish genome. Among these, 35 SNPs could be annotated unambiguously (Table S2). Three SNPs are of particular interest, as they are potentially under selection and involved in development: i) the collagen alpha-1 (XXVII) chain B (RAD marker 3771), ii) the microtubule-actin cross-linking factor 1 (RAD marker 110970), and iii) the nesprin 1 (RAD marker 123874).

### Selection and parallelism in shape QTL: wild Whitefish

A total of 19 of the 138 SNPs that were associated with body shape QTL were significant outliers between dwarf and normal species for at least one lake after corrections for multiple tests with the single-locus Fdist analysis, suggesting that their level of divergence between dwarf and normal Whitefish may have been influenced by divergent selection. These were distributed over many linkage groups (Table 3). An average of four outlier loci were found in each lake (four loci in Cliff, seven in East, five in Indian, two in Témiscouata, and two in Webster, Table 3). Only one locus showed parallelism for two lakes (Indian and Webster); LG 12, position 38.2 (RAD marker 69063; Table 3). Two loci separated by only 1.2 cM showed potential divergent selection between dwarf and normal Whitefish from two lakes (Cliff and Indian); LG 21, position 81 and 82.2 (RAD marker 107544 and 37687; Table 3).

**Table 3:** Description of SNP markers potentially under divergent selection in wild populations of Lake Whitefish as revealed by Fdist analysis

SNP	Associated QTL		*F*_ST_ of Loci Under Potential Divergent Selection in Lake				
	LG	Position, cM	Cliff (0.22)	East (0.03)	Indian (0.11)	Témiscouata (0.01)	Webster (0.05)
46086	1	20.0	−	−	0.49	−	−
78628	4	80.3	−	−	−	0.13	−
147541	4	92.3	−	0.22	−	−	−
107600	6	16.0	−	0.27	−	−	−
110970	10	37.0	−	0.24	−	−	−
132516	12	27.1	−	0.26	−	−	−
**69063**	12	38.2	−	−	**0.67**	−	**0.37**
33001	16	60.4	0.96	−	−	−	−
123874	17	51.5	−	−	−	−	0.31
**107544**	21	81.0	−	−	**0.67**	−	−
**37687**	21	82.2	**0.39**	−	−	−	−
1790	25	29.1	−	0.17	−	−	−
35278	26	36.7	−	−	0.44	−	−
88462	28	38.3	−	0.27	−	−	−
101670	31	10.8	0.77	−	−	−	−
3771	31	14.9	−	−	−	0.27	−
74955	34	36.2	0.78	−	−	−	−
45298	36	19.0	−	0.28	−	−	−
71661	39	21.9	−	−	0.68	−	−

The LG as defined in Gagnaire *et al.* (2013a) and position of the body shape−associated QTL are indicated. In addition, *F*_ST_ values between dwarf and normal Whitefish in the lake in which potential selection was detected are indicated. The mean *F*_ST_ value for divergence between dwarf and normal Whitefish for 3438 SNPs is indicated in parentheses under the lake names. In bold, a marker (69063) that shows potential genetic parallelism between Indian and Webster species pairs and two others (107544 and 37687) physically ’close’ (1.2 cM) on the genetic map that could represent another case of genetic parallelism between Cliff and Indian species pairs. SNP, single-nucleotide polymorphism; QTL, quantitative trait loci; LG, linkage group.

**Figure 4:**
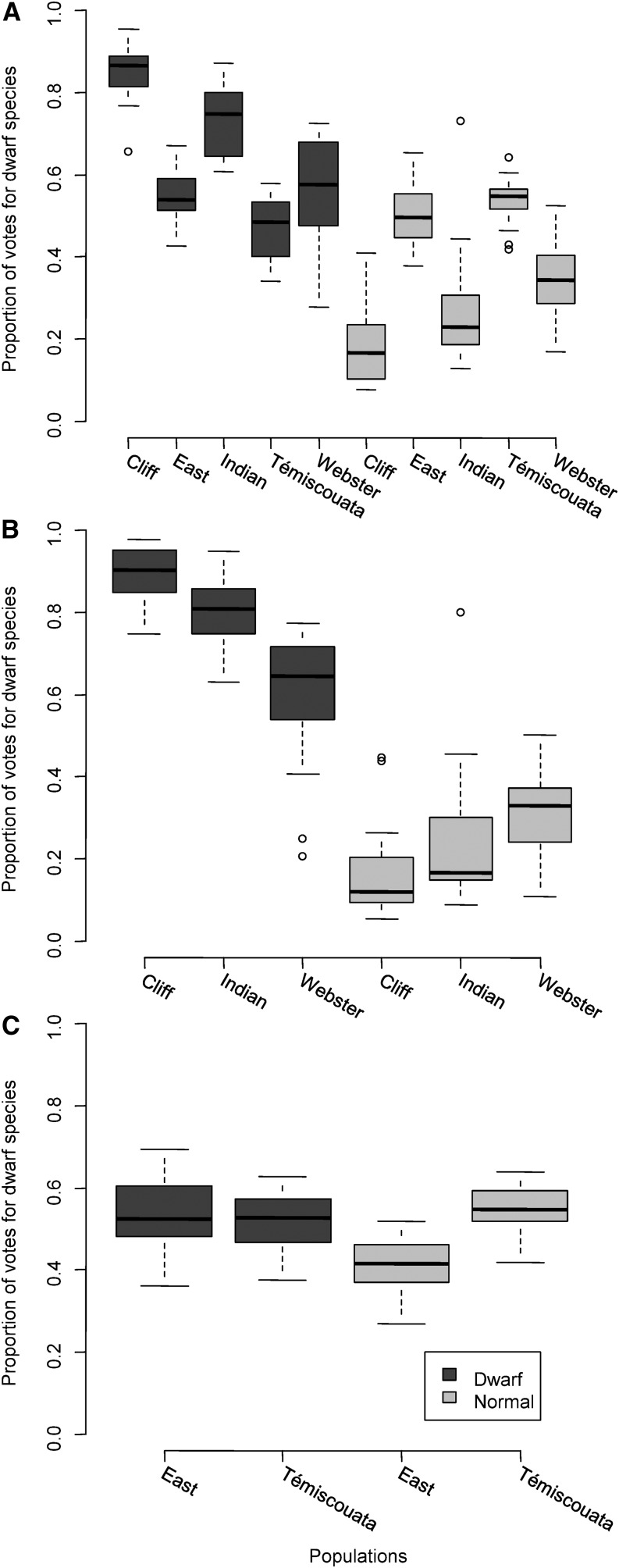
Boxplot of the proportion of “votes” for Whitefish from dwarf populations (dark gray) and normal populations (light gray) (x-axis) for the dwarf species group (y-axis) obtained via 1,000,000 trees produced with random forest analyses on 138 shape-related QTL. A “vote” refers the classification of an individual by a single tree. (A) All 10 populations from five lakes—classification error rate of 24.3%; (B) six of these populations from three lakes (Cliff, Indian, and Webster)—classification error rate of 5.9% and (C) the four remaining populations from two lakes (East and Témiscouata)—classification error rate of 40.5%.

The polygenic Random Forest algorithm produced on the five lakes identified 33 important markers. The proportion of votes differentiated individuals of both species in Cliff, Indian, and Webster lakes, but not in East and Témiscouata lakes (Figure 4A). This finding suggests that these three lakes shared a common genetic architecture underlying morphologic differentiation between dwarf and normal Whitefish (*i.e.*, similar genotypes on the 138 shape QTL allowing one to differentiate the two species) that is not shared with the other two lakes. The overall Random Forest classification error rate was 24.3% and was concentrated mainly in East and Témiscouata lakes according to the proportion of votes (Figure 4A). Given this, we ran the Random Forest algorithm two more times; i) on the species pairs from Cliff, Indian, and Webster lakes, to test whether the classification error rate will decrease when considering these three lakes only and ii) on the species pairs from East and Témiscouata to test whether genetic parallelism specific to these two species pairs is present. We found a classification error rate of only 5.9% when considering Cliff, Indian and Webster lakes and 38 important markers (Figure 4B). The mean *F*_ST_ for these 38 important markers was low, averring 0.08. Moreover, the mean *F*_ST_ between dwarf and normal Whitefish from Cliff, Indian, and Webster lakes was significantly greater for RAD markers under potential divergent selection (n = 10; four from Cliff, four from Indian, one from Webster, and one share between Indian and Webster) than for the 38 important markers among the three lakes (*F*_ST_ = 0.32 *vs.* 0.08; t = −4.94, P-value < 0.001). Only three markers were shared between the two approaches. Yet, there was a trend for the mean PVE for individual marker to be greater for the important markers (PVE = 4.85%) than for the outliers potentially under divergent selection (PVE = 3.07%), although this difference was not statistically significant (t = 1.58, P-value = 0.131). In addition, important markers obtained from Indian and Webster species pairs allowed assigning correctly 100% of the individuals from Cliff lake. Similarly, analysis from Cliff and Webster species pairs assigned correctly 95% of the individuals from Indian lake and analysis from Cliff and Indian species pairs correctly assigned 85% of the individuals from Webster lake. Thus, an average of 93.3% of individuals were correctly assigned when not including a given species pair for identifying important markers. In contrast, genetic parallelism appears to be absent between East and Témiscouata lakes with a classification error rate of 40.5% (Figure 4C, number of important markers = 4, see Table S1).

## Discussion

We found evidence for parallel body shape differentiation among dwarf and normal Lake Whitefish in five different lakes. In all cases dwarf fish have larger eyes, more slender bodies, and longer tails compared with the benthic normal species. These differences are consistent with expectations regarding adaptation to the limnetic niche (Langerhans and Reznick 2010; Willacker *et al.* 2010). We also identified QTL underlying variation in body shape, thus revealing a genetic basis for shape variation, although this does not exclude by any mean an environmental effect on shape variation as well (in this section, to follow) as this would be the case for most phenotypic traits in any species. Moreover, the polygenic approach used to detect a group of covarying markers that differentiated the two species showed evidence of genetic parallelism in three of the five lakes (Cliff, Indian, and Webster) we studied. A low mean *F*_ST_ = 0.08 of these covarying markers suggests a recent polygenic adaptation and high percentages of correct assignation, when a species pairs was not included to identify important markers, also support the occurrence of genetic parallelism underlying phenotypic parallelism among these three lakes. In contrast, there was little evidence for genetic parallelism with the single-locus approach that detects outlier under potential divergent selection, as only two genomic regions associated with a body shape QTL showed parallelism between at least two of the five lakes (LG 12, Position 38.2: Indian and Webster and LG 21, Position 81.0 to 82.2: Cliff and Indian). Interestingly, no genetic parallelism was observed between East and Témiscouata Lakes, suggesting a possibility of three different genetic paths that have led to the evolution of dwarf-normal Whitefish species pairs in this system (1: Cliff, Indian and Webster; 2: East and 3: Témiscouata).

Two non-mutually exclusive hypotheses may explain the observation of variation in the extent of parallelism between the phenotype and the genotype: i) a polygenic architecture in which different groups of loci contribute to phenotypic differentiation among lakes and ii) environmental influences on the phenotype which differ among lakes leading to phenotypic plasticity. The high number of shape-related QTL found in the BC family support the polygenic architecture hypothesis since all fish were reared in identical conditions. In addition, quantitative genetic theory predicts that polygenic methods may be better at exploring the genetic basis of complex traits such as body shape (Le Corre and Kremer 2012). For instance, as observed in Bourret *et al.* (2014) in Atlantic salmon, the polygenic analysis revealed genetic parallelism associated with differential mortality whereas single-locus outlier method did not, and recent studies using Random Forest algorithm show high power to detect polygenic selection (Brieuc *et al.* 2015; Pavey *et al.* 2015). Body shape differentiation in Lake Whitefish may therefore be explained by a complex polygenic architecture and the combined effects of relatively modest allelic frequency changes, as reflected by the low mean *F*_ST_ observed at the markers identified by the Random Forest analysis. As mentioned previously, the results of these analyses suggest that phenotypic differentiation observed in this study may have evolved through three different patterns of subtle allelic frequency changes in i) Témiscouata, ii) East, and iii) Cliff/Indian/Webster lakes. Historic contingency offers an explanation for the presence of these three potential genetic pathways found for body shape differentiation as Cliff, Indian, and Webster lakes are closer geographically. In addition, their environments may have been more similar when independent secondary contacts occurred between the Acadian and Atlantic glacial lineages in those lakes (Bernatchez and Dodson 1990). Evidence also suggests that the sympatric pairs from East Lake may have evolved from a single glacial lineage, unlike the other lakes studied (Pigeon *et al.* 1997; Rico *et al.* 2013). Moreover, gene flow between Témiscouata species pairs is the highest documented among the five lakes (Gagnaire *et al.* 2013b; Rico *et al.* 2013) and could result in a loss of genetic differentiation at some loci that are related to body shape differentiation. In addition to a polygenic architecture, phenotypic plasticity evolves following adaptive divergence in new environments and could therefore contribute to an increase in phenotypic parallelism among lakes, in addition to the polygenic basis for body shape that we have identified here (Aubin-Horth and Renn 2009; Angers *et al.* 2010; Pfennig *et al.* 2010, Morris *et al.* 2014).

We identified a total of 138 loci (including 26 at genome-wide significance) that affected at least one body shape coordinate. On average, each phenotypic trait is associated with more than five QTL (between 3 and 11). These data indicate a highly polygenic architecture for body shape in Lake Whitefish which agrees i) with our previous findings of a polygenic basis underlying the expression of other complex ecologically important traits in Lake Whitefish such as growth rate, age at maturity, and behavior (Rogers and Bernatchez 2007; Filteau *et al.* 2013; Gagnaire *et al.* 2013a). This is also congruent with other studies revealing polygenic architecture of shape and other morphometrics (Albert *et al.* 2008; Cooper *et al.* 2011; Wang *et al.* 2011; Hecht *et al.* 2012; Rogers *et al.* 2012; Franchini *et al.* 2014). Also, it must be considered that QTL detected in this study resulted from crossing a normal Whitefish from Aylmer Lake not involved in wild populations considered in this study. This is because it was not possible to obtain wild sexually mature normal fish from the studied populations. Therefore, some QTL found in this study could be cross specific and not segregate in other populations. However, we are confident that this bias is not major in the detection of parallelism among our samples. Namely, previous QTL studies involving other phenotypic traits in this system (*e.g.*, growth) using this same cross and comparing with natural populations revealed the occurrence of parallelism for the same segregating QTL (Rogers and Bernatchez 2007). If anything, this render our interpretations of parallelism more conservative given that we are detecting parallelism despite the possible confounding effect of variation in genetic architecture among populations. To our defense also, very few studies have actually built genetic maps for each individual wild populations being investigated in similar types of studies as performed here.

Functional morphology provides clear *a priori* expectations for the effects of changes in body shape. A slender, fusiform body shape and caudal peduncle diminish drag and can reduce the energetic costs of prolonged swimming in fishes (Webb 1982, 1984; Langerhans and Reznick 2010; Walker 2010; Willacker *et al.* 2010). Thus, we predicted that the dwarf Whitefish species would evolve a more slender shape. This is because the dwarf Whitefish forages on zooplankton in open water, which shows greater variation in abundance and distribution in lakes in comparison to benthic prey (Del Giorgio and Gasol 1995). Moreover, the Lake Trout (*Salvelinus namaycush*), common in all lakes surveyed, is a main predator of Lake Whitefish (Scott and Crossman 1973; Chouinard *et al.* 1996), and is more likely to chase the dwarf Whitefish for longer distances in the greater luminosity of the limnetic environment (Vogel and Beauchamp 1999). Previous studies in controlled experimental conditions also showed that dwarf Whitefish exhibit genetically-based differences in swimming behavior in accordance with expectations based on their ecology (Rogers *et al.* 2002; Rogers and Bernatchez 2007). However, the dwarf Whitefish caudal peduncle depth was not more slender than in the normal species. Since a stout peduncle better powers “fast-start burst swims” (Webb 1982, 1984; Langerhans and Reznick 2010; Walker 2010), it is possible that dwarf Whitefish cannot maximize drag reduction because this may come at the cost of burst swimming performance. Therefore, a slender body but a similar peduncle depth in comparison with the normal species could represent a trade-off to minimize the costs of foraging and predation risk and maintain adequate burst and prolonged swimming capacities. This hypothesis will need to be tested by measuring swimming performance in dwarf and normal Whitefish in future experiments.

Among the annotated SNPs associated with body shape QTL, three are of particular interest because they are found in genes with well-known functions in vertebrate development and are potentially under divergent selection: MACF1, Col27a1b, and Nesprin-1. The microtubule-actin cross-linking factor 1 (MACF1; RAD marker 110970) is highly expressed in mouse neurons and skeletal muscle during embryonic development (Barsi *et al.* 2005; Chen 2006; Koo *et al.* 2007) and regulates animal-vegetal polarity in the zebrafish oocyte (Gupta *et al.* 2010). It has a role in the activation of the canonical Wnt signaling pathway (Wnt/β-catenin pathway), which is involved in embryo patterning and the determination of cell fate through transcriptional activation of target genes, among other functions (Chen 2006). The collagen alpha-1(XXVII) chain B (Col27a1b) (RAD marker 3771) is expressed in the notochord and cartilage of zebrafish embryo. The knockout of this protein results in curvature of the notochord and scolioses in zebrafish embryos. It is thus involved in notochord morphogenesis and axial skeletogenesis (Christiansen *et al.* 2009). Finally, Nesprin-1 (RAD marker 123874) is involved in muscle cell differentiation, nuclear positioning and anchorage (Zhang *et al.* 2001, 2010). Nesprin-1 knockout in mice results in a decrease in survival rates, growth, and exercise capacity and increased variability in body weight (Zhang *et al.* 2010). All of these traits are potentially linked with the phenotypic differentiation observed between Lake Whitefish wild species pairs. As mentioned previously, these genes were all under potential divergent selection in at least one lake (MACF1: East; Col27a1b: Témiscouata; Nesprin-1: Webster) and they are also mistranscribed in malformed hybrid backcrossed Whitefish embryos relative to pure parental forms (Dion-Côté *et al.* 2014). The role of these candidate genes during development suggests that shape could be genetically determined early during the embryonic stage in Lake Whitefish. This finding is supported by the presence of a pronounced shape differentiation between young juveniles of both species raised in common garden (M. Laporte, unpublished data). Of the 35 annotated QTL, 11 corresponded to transposable elements. This is noteworthy as transposable elements are recognized as “powerful facilitators of evolution and phenotypic diversity” (Oliver and Greene 2009) through their transcriptional impact on gene networks (Feschotte 2008). Hence, our results raise the possibility that transposable elements could affect the expression of key genes that are responsible for fish shape determination in the Lake Whitefish. Incidentally, a recent study in Lake Whitefish revealed a reactivation of the expression of transposable elements in backcrossed hybrids which is associated with phenotypic malformation of hybrids during their early development (Dion-Côté *et al.* 2014). Although the effects of these alleles on fish shape in Lake Whitefish embryos are unknown, these proteins and transposable elements are candidates for future studies aiming to decipher the molecular basis of shape differentiation in the Lake Whitefish system.

In conclusion, we found a pronounced pattern of phenotypic parallelism in body shape between dwarf and normal Whitefish in five different species pairs, which fitted the *a priori* predictions based on the known ecology of dwarf and normal Lake Whitefish. This brings support to the role for natural selection in driving body shape differences between these forms. Body shape was also shown to be under polygenic control and genotypic parallelism appears to be present in three of the five lakes as revealed by a polygenic analytical approach. In comparison, the single-locus approach identified very limited cases of genetic parallelism in body shape. We also identified candidate genes to be further investigated and that could contribute to fish shape differentiation based on functional annotation. Overall, our results support the view that both multiple genetic routes and genetic parallelism can occur during the evolution of fish shape to produce phenotypically similar adaptive changes in body shape when facing similar environmental challenges.

## Supplementary Material

Supporting Information

Blog post: Multiple Paths to the Same Result: Parallel Evolution in Lake Whitefish

Corrigendum
