# Supplementary material for: RAD-QTL Mapping Reveals Both Genome-Level Parallelism and Different Genetic Architecture Underlying the Evolution of Body Shape in Lake Whitefish (Coregonus clupeaformis) Species Pairs
Source: G3 (Bethesda). 2015 May 21;5(7):1481–91. doi: 10.1534/g3.115.019067 (PMC4502382; doi:10.1534/g3.115.019067)
Supplement: Supporting Information [file supp_5_7_1481__index.html]

RAD-QTL Mapping Reveals Both Genome-Level Parallelism and Different Genetic Architecture Underlying the Evolution of Body Shape in Lake Whitefish (Coregonus clupeaformis) Species Pairs — Supporting Information 

# RAD-QTL Mapping Reveals Both Genome-Level Parallelism and Different Genetic Architecture Underlying the Evolution of Body Shape in Lake Whitefish (*Coregonus clupeaformis*) Species Pairs

## Supporting Information for Laporte *et al.*, 2015

**Files in this Data Supplement:**

- Supporting Information - Figure S1 and Tables S1-S3 (PDF, 248 KB)
- Figure S1 - Mean shape comparisons between males (dashed line) and females (solid line) in lab-raised Whitefish crosses. (PDF, 160 KB)
- Table S1 - Summary statistics for the 138 shape-associated QTL including linkage group (LG) and map position in cM, analyses in which the QTL was found (with sex as cofactor (cofactor), without or both), the detection threshold (Linkage group (LG) = 0.01; Genome-wide (GW) = 0.05), LOD, P-value, percent variance explained (PVE), the shape coordinates and shape part affected and the associated SNP with distance from the QTL in cM (if any). (PDF, 141 KB)
- Table S2 - Gene annotations and biological process for QTL associated SNPs, including scaffold genome number (Table S3) with it start hit and end (Pavey *et al.* unpubl. data; Table S3), bit score, blast hit scaffold region and blast hit e-value. (PDF, 128 KB)
- Table S3 - Sequence of the 30 genomes scaffolds from Pavey *et al.* (unpubl. data) that were used for annotation in this study. (.fasta, 256 KB)
