# Supplementary material for: RAD-QTL Mapping Reveals Both Genome-Level Parallelism and Different Genetic Architecture Underlying the Evolution of Body Shape in Lake Whitefish (Coregonus clupeaformis) Species Pairs
Source: G3 (Bethesda). 2015 May 21;5(7):1481–91. doi: 10.1534/g3.115.019067 (PMC4502382; doi:10.1534/g3.115.019067)
Supplement: Supporting Information [file supp_g3.115.019067_019067SI.pdf]

G3 · Genes | Genomes | Genetics

RAD-QTL mapping reveals both genome-level parallelism and different genetic architecture underlying the evolution of body shape in Lake Whitefish (*Coregonus clupeaformis*) species pairs

Martin Laporte*, Sean M. Rogers§, Anne-Marie Dion-Côté*, Eric Normandeau*, Pierre-Alexandre Gagnaire†, Anne C. Dalziel*, Jobran Chebib§, Louis Bernatchez*

* Institut de Biologie Intégrative et des Systèmes (IBIS), Département de Biologie, Université Laval, Pavillon Charles-Eugène-Marchand, Québec, G1V 0A6, Canada

§ Department of Biological Sciences, University of Calgary, 2500 University Drive N.W., Calgary, Alberta, T2N 1N4, Canada

† Institut des Sciences de l'Evolution – Montpellier (ISEM - CNRS UMR5554), SMEL, 2 rue des Chantiers, 34200 Sète, France

Corresponding author:
Martin Laporte
Institut de Biologie Intégrative et des Systèmes (IBIS), Département de Biologie, Université Laval, 1030 avenue de la Médecine, Québec, G1V 0A6, Québec, Canada.
Téléphone: (418) 656-2131 #8455
e-mail: uni.mlaporte@gmail.com

DOI: 10.1534/g3.115.019067

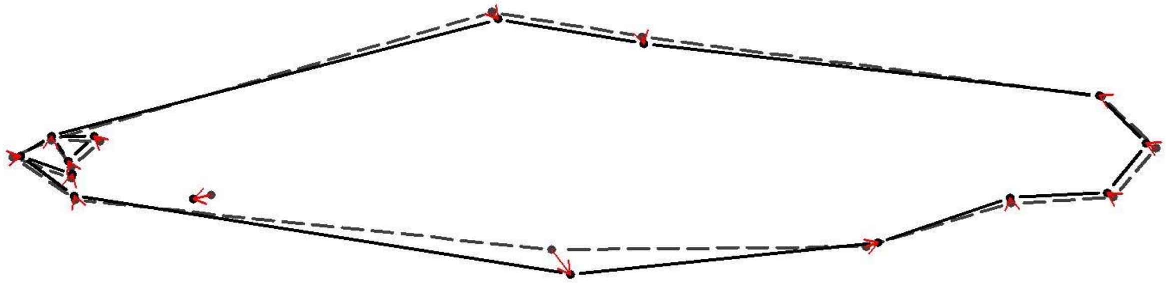

Figure S1 Mean shape comparisons between males (dashed line) and females (solid line) in lab-raised Whitefish crosses. The red arrows show the direction of shape change from male to female. The difference between mean shapes of both treatments was multiplied by two for clarity.

Table S1 Summary statistics for the 138 shape-associated QTL including linkage group (LG) and map position in cM, analyses in which the QTL was found (with sex as cofactor (cofactor), without or both), the detection threshold (Linkage group (LG) = 0.01; Genome-wide (GW) = 0.05), LOD, P-value, percent variance explained (PVE), the shape coordinates and shape part affected and the associated SNP with distance from the QTL in cM (if any). The 1.5 LOD support is the interval, in cM, in which the LOD score is within 1.5 units of its maximum.

#QTL	LG	Position (cM)	1.5 LOD (cM)	PVE	Threshold	LOD	P-value	Shape coord	Nearest marker	QTL distance
**1**	1	16.0	14.3-16.9	2.99	LG	4.73	0.007	x8	19208	0.1
1	1	16.0	13.0-16.9	2.99	GW	5.26	<0.001	x2		
**2**	1	20.0	19.2-21.9	3.58	LG	4.98	0.001	y12	46086*[ab]	0.2
**3**	1	32.1	31.5-33.3	4.80	LG	4.36	0.007	x10	21285	0.0
**4**	2	11.0	8.0-14.0	7.41	LG	4.55	0.005	y11	138429[a]	0.6
**5**	2	12.6	11.0-15.5	2.79	LG	4.01	0.01	y15	6903	0.0
**6**	2	26.0	20.4-32.0	4.22	LG	4.50	0.004	x6	27266	0.2
**7**	2	27.0	25.5-30.9	8.10	GW	6.08	0.001	y5	115021	0.0
	2	27.0	25.5-30.9	9.64	GW	5.69	<0.001	y6		
**8**	2	29.8	26.6-30.9	7.47	LG	4.64	0.004	x9	46661	0.0
**9**	2	33.1	31.0-34.3	9.49	LG	4.64	0.002	y7	39926	0.0
	2	33.1	31.0-39.8	6.09	LG	4.27	0.006	y8		
**10**	2	35.7	32.9-36.3	1.75	LG	4.67	0.009	x13	99310[ab]	0.0
**11**	2	36.3	34.0-38.0	6.65	LG	5.03	0.003	x8	90861	
**12**	2	38.6	37.5-40.7	0.46	LG	4.76	0.004	y4	123465	0.0
**13**	3	2.0	0.0-10.0	1.08	LG	4.77	0.002	x8	81054	1.2
**14**	3	6.4	0.0-7.9	0.59	LG	4.16	0.01	y12	87937	0.0
**15**	3	7.9	0.0-10.0	2.10	LG	4.07	0.006	x7	5096	0.0
	3	7.9	0.0-16.8	7.77	LG	4.00	0.01	x6		
**16**	3	9.1	7.0-11.0	1.12	GW	7.11	<0.001	x11	114971	0.0
**17**	3	44.9	40.0-51.6	1.16	LG	3.87	0.008	x10	69007[b]	0.0
**18**	4	26.0	15.0-35.7	1.28	LG	5.17	0.009	y3	33146[b]	2.8
**19**	4	40.5	39.0-45.9	2.73	LG	4.82	0.014	x7	123418	0.0
**20**	4	64.5	63.6-66.0	5.84	LG	4.99	0.003	x2	20896	0.0
**21**	4	70.0	63.6-70.1	7.18	GW	5.68	0.002	x1	64346	0.0
**22**	4	70.7	70.0-72.5	1.41	LG	4.63	0.008	y15	114130[b]	0.0
**23**	4	71.0	70.3-72.5	4.41	LG	4.76	0.009	y9	66793	0.1
**24**	4	79.7	78.7-80.3	5.42	LG	4.82	0.008	x14	88186	0.0

**25**	4	80.3	79.7-81.0	3.55	GW	5.61	0.003	x10	78628*[b]	0.0
**26**	4	92.2	90.1-92.9	3.93	LG	5.18	0.009	y1	147541*	0.0
**27**	4	106.0	104.1-106.6	1.95	GW	5.75	0.001	y2	125074	0.2
**28**	4	107.0	105.8-107.0	0.26	GW	5.55	0.003	x9	267	0.1
	4	107.0	105.2-106.6	4.29	GW	5.73	<0.001	y10		
**29**	5	17.5	15.0-21.8	5.05	LG	4.98	0.006	y3	51058[ab]	0.0
**30**	5	49.6	45.4-50.9	7.68	LG	5.28	0.004	x4	104425	0.0
**31**	5	50.0	48.0-51.2	9.49	LG	5.19	0.003	x5	50194	0.2
**32**	5	51.7	48.8-55.2	8.88	GW	6.10	<0.001	x9	35273	0.0
**33**	5	52.2	51.2-54.0	4.03	LG	5.10	0.004	x8	14817	0.0
**34**	5	68.2	68.1-70.4	1.71	LG	4.97	0.006	y12	43047	0.0
**35**	6	16.0	15.2-16.3	7.54	GW	5.30	<0.001	y4	107600*[ab]	0.0
	6	16.0	13.5-16.2	2.23	GW	5.27	<0.001	y5		
	6	16.0	15.2-16.2	11.87	GW	7.55	<0.001	y6		
**36**	7	8.8	8.3-8.9	4.50	LG	4.58	0.003	x1	107557	0.00
	7	8.8	8.3-8.9	7.23	LG	4.99	0.002	x2		
**37**	7	18.0	16.1-19.9	2.12	LG	4.63	0.008	x11	101290	0.0
**38**	8	10.0	0.0-15.0	0.45	LG	4.38	0.005	x5	93783	0.5
**39**	8	10.8	0.0-14.0	2.21	LG	4.24	0.006	y9	151762	0.0
**40**	8	58.4	57.3-60.4	14.72	GW	6.14	0.001	x11	70187[a]	0.0
**41**	9	60.0	57.0-63.0	10.46	LG	4.79	0.002	y15	131325[ab]	0.22
	9	61.0	57.0-63.8	3.85	LG	4.60	0.005	y14		0.78
	9	61.0	57.8-65.0	1.80	LG	4.33	0.009	y2		0.78
**42**	9	83.9	74.0-86.0	1.47	LG	4.71	0.008	y10	23705[b]	0.0
	9	83.9	82.0-87.0	5.82	LG	4.68	0.002	y11		
**43**	10	36.4	35.2-37.9	5.11	LG	4.73	0.005	y14	64568	0.0
**44**	10	37.0	35.9-39.3	1.43	GW	5.52	0.001	y3	110970*	0.1
**45**	11	34.5	31.0-37.0	6.29	LG	4.32	0.003	y13	152398	0.0
**46**	11	35.0	30.1-37.0	7.56	LG	4.56	0.002	y9	46466	0.1
**47**	11	37.9	37.2-48.6	0.64	LG	4.73	0.004	y12	42247[ab]	0.0
**48**	11	60.7	59.0-64.0	8.41	LG	5.14	0.004	x13	8080	0.00
**49**	12	6.9	3.0-8.0	7.29	LG	4.95	0.004	y10	155423[ab]	0.0
**50**	12	8.0	4.0-12.0	1.97	LG	4.37	0.006	y7	23679	0.21
	12	8.0	6.9-12.0	1.44	GW	5.63	0.002	y8		
**51**	12	27.1	27.0-27.8	5.53	GW	5.55	0.001	x1	132516*	0.0
**52**	12	38.2	30.5-38.8	1.18	LG	4.57	0.001	x13	69063*	0.0
**53**	12	54.9	50.7-56.0	6.55	LG	4.38	0.009	x3	893	0.0
**54**	13	20.0	18.3-23.0	1.18	LG	4.40	0.008	y4	126101	1.1
**55**	13	41.3	35.0-45.5	6.39	LG	5.05	0.004	y13	120901	0.0
**56**	13	52.0	43.7-74.0	1.20	LG	4.52	0.005	y1	38077	0.4
**57**	13	54.0	52.4-61.0	0.42	GW	6.31	<0.001	y2	64843	0.0
**58**	13	55.0	52.0-60.0	5.16	GW	7.34	<0.001	x4	146313[ab]	0.8

	13	55.8	52.0-59.0	4.92	LG	5.54	<0.001	x5		0.0
**59**	13	73.0	60.0-87.6	2.12	LG	3.97	0.01	x2	18689	0.2
**60**	14	11.6	9.0-17.0	2.63	LG	3.91	0.008	y2	19673	0.0
**61**	14	18.2	14.7-19.6	1.81	LG	4.97	0.001	x13	123800	0.0
**62**	15	29.9	24.2-32.7	2.37	LG	4.20	0.009	y12	107006[ab]	0.0
**63**	16	10.1	0.0-23.0	0.79	LG	4.86	0.001	y13	89546	0.0
**64**	16	42.0	36.0-44.0	1.10	LG	3.71	0.01	x1	49883[ab]	0.3
**65**	16	46.1	42.5-47.0	11.81	LG	4.24	0.003	y7	3271[ab]	0.0
	16	46.1	43.0-47.5	10.97	LG	4.29	0.005	y8		
	16	46.1	44.1-47.5	4.96	LG	4.91	0.002	y1		
**66**	16	58.8	54.0-64.0	1.92	LG	4.21	0.006	x9	147458	0.0
**67**	16	60.4	56.8-66.8	2.04	LG	3.76	0.009	x8	33001*	0.0
**68**	16	63.1	57.0-66.8	5.85	LG	3.84	0.01	x7	132794	0.0
**69**	16	78.2	68.6-89.0	1.26	LG	3.90	0.004	y12	100560[ab]	0.0
**70**	17	37.8	34.8-42.7	8.01	LG	4.01	0.006	x12	13798	0.0
**71**	17	44.2	43.1-50.1	12.42	LG	4.99	<0.001	x13	145673[b]	0.0
**72**	17	51.5	49.3-52.3	5.87	LG	4.21	0.005	y15	123874[ab]	0.0
	17	51.5	48.0-52.3	4.76	LG	5.60	0.001	y2		
**73**	17	51.7	48.0-52.9	2.59	LG	4.56	0.002	y1	58078*	0.0
**74**	17	51.8	46.0-52.3	2.90	LG	5.13	0.002	x4	74080[b]	0.0
**75**	18	22.7	21.0-24.8	6.51	GW	7.54	<0.001	y3	41690	0.0
	18	23.0	18.0-29.0	4.72	LG	4.28	0.005	y15		0.3
**76**	19	19.0	14.0-26.0	2.22	LG	4.67	0.01	y12	9185[b]	0.9
**77**	19	22.0	21.2-24.9	7.43	LG	4.81	0.005	x12	128619	0.0
**78**	19	29.7	27.0-31.9	3.52	LG	4.08	0.01	x13	70939	0.0
**79**	21	61.9	49.5-69.9	0.97	LG	4.01	0.009	y4	136924	0.0
**80**	21	72.6	71.0-73.5	9.54	LG	5.88	<0.001	y8	127803[ab]	0.0
**81**	21	77.8	75.0-82.2	3.90	LG	4.37	0.001	x12	8717[ab]	0.0
**82**	21	81.0	78.0-84.0	2.88	GW	5.67	<0.001	y12	107544*[ab]	0.8
**83**	21	82.2	77.0-86.0	10.15	GW	5.90	0.002	y10	37687*	0.0
**84**	23	2.6	2.0-4.0	6.38	LG	4.31	0.005	x2	38781	0.0
**85**	23	11.0	5.0-15.6	4.93	LG	3.74	0.009	y5	29770	1.9
**86**	23	30.0	26.6-32.8	2.40	LG	4.15	0.003	y6	131558[a]	0.2
**87**	24	54.5	53.3-58.5	1.12	LG	5.14	0.002	x9	81546[ab]	0.0
**88**	25	26.0	18.2-30.7	14.59	LG	4.94	0.002	x7	33027	0.6
**89**	25	29.1	28.2-30.0	3.77	LG	4.76	0.005	x3	1790*	0.0
**90**	26	6.0	0.0-10.0	2.89	LG	3.99	0.009	y6	20067	1.2
**91**	26	36.7	36.5-37.9	2.43	GW	5.40	<0.001	x15	35278*	0.0
**92**	26	80.0	66.0-81.0	1.48	LG	4.74	0.007	y12	65665[ab]	0.9
**93**	27	0.0	0.0-15.0	4.32	LG	4.55	0.004	y1	73904	0.0
**94**	27	38.9	35.6-41.4	11.00	GW	5.28	0.002	x3	80526	0.0

**95**	27	64.6	60.0-64.6	4.37	GW	5.90	<0.001	y10	48708	0.3
	27	64.6	58.0-64.6	5.08	LG	4.62	0.002	y11		0.3
**96**	28	38.0	33.0-43.0	4.73	GW	5.93	<0.001	y2	88462*	0.3
	28	38.3	32.0-42.3	3.33	LG	4.23	0.004	y7		0.0
	28	38.3	36.3-39.6	3.84	LG	4.33	0.003	y8		
	28	38.3	33.2-39.6	1.80	GW	6.06	<0.001	y1		
**97**	29	9.0	8.0-13.0	1.81	GW	5.35	0.002	x14	12305	0.1
	29	9.1	8.00-14.00	1.46	LG	3.98	0.009	x15		0.0
	29	11.0	8.0-16.0	7.16	LG	4.47	0.006	y9		1.9
**98**	29	17.5	16.1-18.1	6.12	LG	4.30	0.004	x3	66525	0.0
**99**	29	29.0	27.0-30.5	5.16	LG	5.02	0.001	x9	109751	0.1
**100**	29	44.0	41.2-46.0	9.04	LG	4.81	0.004	y6	41781[ab]	0.2
**101**	29	54.3	49.2-56.0	3.04	LG	4.15	0.006	x4	27081	0.0
**102**	31	10.8	9.0-16.9	2.30	LG	4.48	0.005	x7	101670 *0.0	
**103**	31	12.2	10.0-13.3	7.77	LG	4.54	0.01	x14	147625	0.0
	31	12.2	9.3-16.9	8.76	GW	5.66	<0.001	x5		
**104**	31	14.9	9.3-16.2	11.19	LG	4.42	0.005	x6	3771*[ab]	0.0
**105**	31	16.2	11.0-23.0	14.35	LG	4.89	0.002	y6	27954	
**106**	31	69.0	65.6-70.6	7.36	LG	4.54	0.007	y3	69815[a]	0.1
	31	69.1	68.1-71.9	12.37	LG	5.13	0.002	y14		0.0
**107**	32	24.4	21.0-27.7	6.29	LG	3.93	0.005	x4	125971[ab]	0.0
	32	24.4	21.0-31.00	2.51	LG	3.78	0.01	x5		
**108**	32	30.0	27.0-34.9	4.60	LG	4.68	0.004	x11	97205	0.0
	32	30.0	29.2-34.0	9.30	LG	4.41	0.006	x12		
	32	30.0	27.0-31.0	1.54	LG	4.06	0.009	y12		
**109**	33	47.0	44.3-63.0	2.67	LG	4.28	0.009	x2	153833[ab]	0.8
**110**	34	6.9	6.0-11.4	10.18	LG	4.13	<0.001	x1	11005	0.0
**111**	34	11.7	10.2-13.1	7.75	LG	3.81	0.008	y13	155701[ab]	0.0
**112**	34	21.9	20.0-22.4	3.23	LG	4.30	0.006	x15	66224	0.0
**113**	34	30.0	27.3-40.0	8.09	LG	4.27	0.005	y7	81895	0.0
	34	30.0	27.7-73.6	5.37	LG	4.32	0.002	y8		
**114**	34	36.2	33.0-73.6	0.47	LG	4.43	0.006	y2	74955*	0.0
**115**	34	41.3	34.3-73.6	1.34	LG	4.15	0.006	y1	103362	0.0
**116**	35	43.8	42.0-44.8	0.98	LG	4.76	0.001	y13	31050[ab]	0.0
**117**	36	6.2	2.0-6.9	0.51	LG	3.87	0.008	y15	64089	0.0
**118**	36	10.0	0.0-14.0	3.18	LG	4.08	0.004	x14	78917	1.3
**119**	36	15.4	13.0-19.0	6.57	LG	3.96	0.006	y1	9528	0.0
	36	15.4	13.0-17.0	2.36	LG	3.96	0.009	y2		
**120**	36	18.9	18.1-19.4	1.25	LG	4.18	0.008	x9	45298*	0.0
**121**	36	23.5	21.9-25.0	8.32	LG	4.50	0.006	x3	114899[b]	0.0
**122**	36	31.4	30.5-42.6	5.29	LG	4.25	0.003	x7	60769[a]	0.0

**123**	37	46.9	29.4-50.5	1.45	LG	4.22	0.01	x9	60023	0.0
**124**	37	56.0	52.4-64.0	1.08	LG	4.34	0.007	x5	136870	0.7
**125**	37	75.3	58.1-75.3	2.37	LG	5.05	<0.001	y1	28863	0.0
**126**	38	1.0	0.0-18.9	2.36	LG	3.98	0.005	y5	138235	1.0
**127**	38	24.9	18.8-31.5	14.34	LG	4.24	0.002	x7	8546[ab]	0.0
**128**	38	30.4	27.9-32.0	16.43	GW	5.69	<0.001	x10	102431[b]	0.0
**129**	38	42.2	37.2-46.0	9.56	GW	5.46	<0.001	y3	21462	0.0
	38	42.2	41.4-44.0	13.03	GW	5.68	<0.001	y4		
**130**	39	2.0	0.0-5.8	1.55	LG	4.35	0.005	x3	6501	1.5
**131**	39	9.2	5.0-9.7	0.98	LG	5.15	0.001	x8	28896[ab]	0.0
**132**	39	14.0	12.1-15.7	4.25	LG	5.23	0.001	x5	67121[ab]	0.0
**133**	39	14.4	12.1-15.7	0.92	LG	4.43	0.007	x7	60846[a]	0.0
	39	14.4	12.1-18.0	4.13	LG	3.76	0.009	x6		
**134**	39	15.0	11.2-15.7	2.15	LG	4.63	0.004	x4	99974[b]	0.0
**135**	39	18.8	17.0-23.9	1.49	LG	3.69	0.008	x13	86377[#]	0.0
**136**	39	21.9	21.1-22.6	0.35	LG	4.41	0.006	x9	71661*	0.0
**137**	39	26.7	23.4-30.0	1.13	LG	3.98	0.007	y15	11245	0.0
**138**	40	11.0	2.0-14.6	2.15	LG	4.66	0.002	x1	98245	2.1

* The 19 associated SNP that were found to be potentially under divergent selection in at least one lake

[#] The 13 associated SNP with annotation

[a] The 33 associated SNP that were found to be important markers with RandomForest ran on all five lakes

[b] The 38 associated SNP that were found to be important markers with random forest ran on Cliff, Indian and Webster lakes only.

Table S2 Gene annotations and biological process for QTL associated SNPs, including scaffold genome number (Table S3) with it start hit and end (Pavey *et al.* unpubl. data; Table S3), bit score, blast hit scaffold region and blast hit e-value.

SNP	Scaffold	hit start	hit end	Bit score	Blast hit region	Blast hit	e-value	Annotation	Biol.process
3771*	53738	723	655	91.6	scaffold-53738_10145-13664	gi|166218146|sp|A0MSJ1.1|CRA1B_DANRE	2.00E-21	Collagen alpha-1(XXVII) chain B	Skeletal system development
6501	18019	18515	18582	121	scaffold-18019_12691-13429	gi|74710067|sp|Q6R2W3.1|SCND3_HUMAN	7.00E-20	SCAN domain-containing protein 3	*Unknown*
13798	436174	7187	7120	121	scaffold-436174_513-2580	gi|122145283|sp|Q0IID7.1|DUS10_BOVIN	2.00E-47	Dual specificity protein phosphatase 10	Cellular signalisation
19208	447255	1037	1104	126	scaffold-447255_500-863	gi|123885751|sp|Q0P427.1|PPR35_DANRE	6.00E-14	Protein phosphatase 1 regulatory subunit 35	Cellular signalisation
21462	183364	2807	2758	87.9	scaffold-183364_3863-4414	gi|464864|sp|P35072.1|TCB1_CAEBR	9.00E-36	Transposable element Tcb1 transposase	Transposable element
28896	232830	3491	3424	104	scaffold-232830_2720-3610	gi|74821373|sp|Q95SX7.1|RTBS_DROME	3.00E-17	Probable RNA-directed DNA polymerase from transposon BS	RNA-dependant DNA replication
37687*	26906	1078	1040	67.6	scaffold-26906_1850-4864	gi|141446|sp|P03934.1|TC1A_CAEEL	2.00E-36	Transposable element Tc1 transposase	Transposable element
38077	208871	9749	9682	126	scaffold-208871_1928-3130	gi|172046699|sp|Q86TG7.2|PEG10_HUMAN	6.00E-43	Retrotransposon-derived protein PEG10	Apoptosis and cell differenciation
38781	133600	2165	2232	121	scaffold-133600_588-6816	gi|143018392|sp|Q9HCG7.2|GBA2_HUMAN	1.00E-20	Non-lysosomal glucosylceramidase	Neuronal development
48708	453732	5661	5728	121	scaffold-453732_3049-4594	gi|51702142|sp|Q9D2M8.4|UB2V2_MOUSE	8.00E-24	Ubiquitin-conjugating enzyme E2 variant 2	Transcriptional regulation and DNA repair
					scaffold-453732_6190-6527	gi|464864|sp|P35072.1|TCB1_CAEBR	4.00E-31	Transposable element Tcb1 transposase	Transposable element
49883	279183	2986	3053	126	scaffold-279183_1927-2442	gi|68565602|sp|Q92585.3|MAML1_HUMAN	2.00E-13	Mastermind-like protein 1	Notch signaling pathway
51058	310208	183	116	126	scaffold-310208_102-461	gi|385178693|sp|F1Q7Z7.2|DAAF3_DANRE	3.00E-24	Dynein assembly factor 3, axonemal	Cytoplasm
60023	7589	4537	4604	121	scaffold-7589_379-636	gi|75041428|sp|Q5R7K9.1|EDIL3_PONAB	2.00E-35	EGF-like repeat and discoidin I-like domain-containing protein 3	Cell adhesion
64568	15526	9006	9073	121	scaffold-15526_1724-2397	gi|464864|sp|P35072.1|TCB1_CAEBR	2.00E-19	Transposable element Tcb1 transposase	Transposable element
					scaffold-15526_6444-9312	gi|122069920|sp|Q2KJB1.1|SEP10_BOVIN	3.00E-69	Septin-10	Cell cycle

69063*	54791	5526	5459	121	scaffold-54791_3224-3664	gi|81880352|sp|Q9CPY0.1|MRM2_MOUSE	2.00E-47	rRNA methyltransferase 2, mitochondrial	rRNA methylation
74955*	76647	1518	1452	124	scaffold-76647_2170-5490	gi|83288218|sp|Q53GS7.2|GLE1_HUMAN	3.00E-36	Nucleoporin GLE1	mRNA export
					scaffold-76647_6723-7102	gi|464865|sp|Q04202.1|TCB2_CAEBR	6.00E-20	Transposable element Tcb2 transposase	Transposable element
78628*	78598	5211	5144	126	scaffold-78598_2145-2291	gi|9910620|sp|O43715.1|TRIA1_HUMAN	5.00E-26	TP53-regulated inhibitor of apoptosis 1	Apoptosis
78917	183364	2847	2914	102	scaffold-183364_3863-4414	gi|464864|sp|P35072.1|TCB1_CAEBR	9.00E-36	Transposable element Tcb1 transposase	Transposable element
80526	232830	3500	3567	126	scaffold-232830_2720-3610	gi|74821373|sp|Q95SX7.1|RTBS_DROME	3.00E-17	Probable RNA-directed DNA polymerase from transposon BS	RNA-dependant DNA replication
88186	336919	2690	2757	126	scaffold-336919_2507-2698	gi|81898999|sp|Q8C627.1|F221B_MOUSE	5.00E-16	Protein FAM221B	*Unknown*
93783	334686	526	459	126	scaffold-334686_207-1019	gi|134035372|sp|Q6A085.2|ZN629_MOUSE	1.00E-30	Zinc finger protein 629	Transcriptional regulation
97205	34582	2640	2706	124	scaffold-34582_9401-10219	gi|464864|sp|P35072.1|TCB1_CAEBR	3.00E-37	Transposable element Tcb1 transposase	Transposable element
					scaffold-34582_17105-17316	gi|50403735|sp|P33005.2|KALM_CHICK	2.00E-29	Anosmin-1	Cell adhesion
101670*	183364	2838	2766	97.1	scaffold-183364_3863-4414	gi|464864|sp|P35072.1|TCB1_CAEBR	9.00E-36	Transposable element Tcb1 transposase	Transposable element
110970*	12855	20623	20586	71.3	scaffold-12855_2228-14681	gi|338817941|sp|Q9QXZ0.2|MACF1_MOUSE	1.00E-62	Microtubule-actin cross-linking factor 1	*Multiple*
	32096	8942	9009	121	scaffold-32096_1541-5542	gi|338817989|sp|Q9UPN3.4|MACF1_HUMAN	2.00E-103	Microtubule-actin cross-linking factor 1, isoforms 1/2/3/5	*Multiple*
114899	84221	5438	5371	126	scaffold-78598_3908-8097	gi|145566945|sp|Q2NKQ1.2|SGSM1_HUMAN	3.00E-63	Small G protein signaling modulator 1	Golgi apparatus
					scaffold-84221_5333-6762	gi|82100806|sp|Q8AXC7.1|PGFRA_TAKRU	1.00E-92	Platelet-derived growth factor receptor alpha	Chemotaxis and development
114971	340942	1	61	113	scaffold-340942_931-1155	gi|123792664|sp|Q0V8T9.1|CTP5A_MOUSE	3.00E-21	Contactin-associated protein like 5-1	Cell adhesion
123874*	228433	1365	1432	121	scaffold-228433_10-3697	gi|425906075|sp|Q8NF91.4|SYNE1_HUMAN	2.00E-22	Nesprin-1	Cytoskeleton and organelle
131325	300418	2369	2302	126	scaffold-300418_521-4385	gi|17367145|sp|Q9ULA0.1|DNPEP_HUMAN	6.00E-19	Aspartyl aminopeptidase	Protein metabolism
136924	64513	1788	1721	126	scaffold-64513_1202-1897	gi|75056157|sp|Q9GLY5.1|ITIH3_RABIT	6.00E-37	Inter-alpha-trypsin inhibitor heavy chain H3	Hyaluronan metabolism

	272191	699	766	121	scaffold-272191_683-862	gi|3024056|sp|Q63416.1|ITIH3_RAT	9.00E-17	Inter-alpha-trypsin inhibitor heavy chain H3	Hyaluronan metabolism
138429	232830	3491	3424	99	scaffold-232830_2720-3610	gi|74821373|sp|Q95SX7.1|RTBS_DROME	3.00E-17	Probable RNA-directed DNA polymerase from transposon BS	RNA-dependant DNA replication
147541*	392290	308	375	126	scaffold-392290_2121-2231	gi|75077434|sp|Q4U0T9.1|CSRP3_BOVIN	4.00E-18	Cysteine and glycine-rich protein 3	Cardiac muscle development
155423	31593	2825	2892	121	scaffold-31593_6120-6725	gi|464864|sp|P35072.1|TCB1_CAEBR	6.00E-36	Transposable element Tcb1 transposase	Transposable element

* Annotated SNP with potential divergent selection.

Table S3 Sequence of the 30 genomes scaffolds from Pavey *et al.* (unpubl. data) that were used for annotation in this study.

Available for download at www.g3journal.org/lookup/suppl/doi:10.1534/g3.115.019067/-/DC1
