# Supplementary material for: RAD-QTL Mapping Reveals Both Genome-Level Parallelism and Different Genetic Architecture Underlying the Evolution of Body Shape in Lake Whitefish (Coregonus clupeaformis) Species Pairs
Source: G3 (Bethesda). 2015 May 21;5(7):1481–91. doi: 10.1534/g3.115.019067 (PMC4502382; doi:10.1534/g3.115.019067)
Supplement: Supporting Information [file supp_g3.115.019067_FigureS1.pdf]

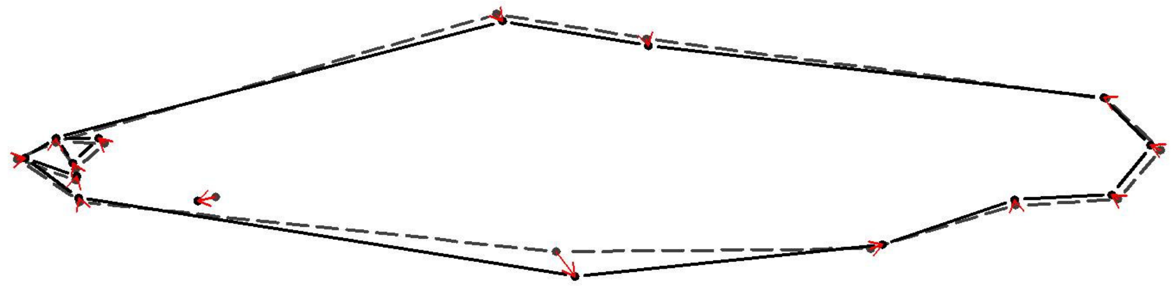

Figure S1 Mean shape comparisons between males (dashed line) and females (solid line) in lab-raised Whitefish crosses. The red arrows show the direction of shape change from male to female. The difference between mean shapes of both treatments was multiplied by two for clarity.
