# Supplementary material for: RAD-QTL Mapping Reveals Both Genome-Level Parallelism and Different Genetic Architecture Underlying the Evolution of Body Shape in Lake Whitefish (Coregonus clupeaformis) Species Pairs
Source: G3 (Bethesda). 2015 May 21;5(7):1481–91. doi: 10.1534/g3.115.019067 (PMC4502382; doi:10.1534/g3.115.019067)
Supplement: Supporting Information [file supp_g3.115.019067_TableS2.pdf]

Table S2 Gene annotations and biological process for QTL associated SNPs, including scaffold genome number (Table S3) with it start hit and end (Pavey *et al.* unpubl. data; Table S3), bit score, blast hit scaffold region and blast hit e-value.

SNP	Scaffold	hit start	hit end	Bit score	Blast hit region	Blast hit	e-value	Annotation	Biol.process
3771*	53738	723	655	91.6	scaffold-53738_10145-13664	gi|166218146|sp|A0MSJ1.1|CRA1B_DANRE	2.00E-21	Collagen alpha-1(XXVII) chain B	Skeletal system development
6501	18019	18515	18582	121	scaffold-18019_12691-13429	gi|74710067|sp|Q6R2W3.1|SCND3_HUMAN	7.00E-20	SCAN domain-containing protein 3	*Unknown*
13798	436174	7187	7120	121	scaffold-436174_513-2580	gi|122145283|sp|Q0IID7.1|DUS10_BOVIN	2.00E-47	Dual specificity protein phosphatase 10	Cellular signalisation
19208	447255	1037	1104	126	scaffold-447255_500-863	gi|123885751|sp|Q0P427.1|PPR35_DANRE	6.00E-14	Protein phosphatase 1 regulatory subunit 35	Cellular signalisation
21462	183364	2807	2758	87.9	scaffold-183364_3863-4414	gi|464864|sp|P35072.1|TCB1_CAEBR	9.00E-36	Transposable element Tcb1 transposase	Transposable element
28896	232830	3491	3424	104	scaffold-232830_2720-3610	gi|74821373|sp|Q95SX7.1|RTBS_DROME	3.00E-17	Probable RNA-directed DNA polymerase from transposon BS	RNA-dependant DNA replication
37687*	26906	1078	1040	67.6	scaffold-26906_1850-4864	gi|141446|sp|P03934.1|TC1A_CAEEL	2.00E-36	Transposable element Tc1 transposase	Transposable element
38077	208871	9749	9682	126	scaffold-208871_1928-3130	gi|172046699|sp|Q86TG7.2|PEG10_HUMAN	6.00E-43	Retrotransposon-derived protein PEG10	Apoptosis and cell differenciation
38781	133600	2165	2232	121	scaffold-133600_588-6816	gi|143018392|sp|Q9HCG7.2|GBA2_HUMAN	1.00E-20	Non-lysosomal glucosylceramidase	Neuronal development
48708	453732	5661	5728	121	scaffold-453732_3049-4594	gi|51702142|sp|Q9D2M8.4|UB2V2_MOUSE	8.00E-24	Ubiquitin-conjugating enzyme E2 variant 2	Transcriptional regulation and DNA repair
					scaffold-453732_6190-6527	gi|464864|sp|P35072.1|TCB1_CAEBR	4.00E-31	Transposable element Tcb1 transposase	Transposable element
49883	279183	2986	3053	126	scaffold-279183_1927-2442	gi|68565602|sp|Q92585.3|MAML1_HUMAN	2.00E-13	Mastermind-like protein 1	Notch signaling pathway
51058	310208	183	116	126	scaffold-310208_102-461	gi|385178693|sp|F1Q7Z7.2|DAAF3_DANRE	3.00E-24	Dynein assembly factor 3, axonemal	Cytoplasm
60023	7589	4537	4604	121	scaffold-7589_379-636	gi|75041428|sp|Q5R7K9.1|EDIL3_PONAB	2.00E-35	EGF-like repeat and discoidin I-like domain-containing protein 3	Cell adhesion
64568	15526	9006	9073	121	scaffold-15526_1724-2397	gi|464864|sp|P35072.1|TCB1_CAEBR	2.00E-19	Transposable element Tcb1 transposase	Transposable element
					scaffold-15526_6444-9312	gi|122069920|sp|Q2KJB1.1|SEP10_BOVIN	3.00E-69	Septin-10	Cell cycle

69063*	54791	5526	5459	121	scaffold-54791_3224-3664	gi|81880352|sp|Q9CPY0.1|MRM2_MOUSE	2.00E-47	rRNA methyltransferase 2, mitochondrial	rRNA methylation
74955*	76647	1518	1452	124	scaffold-76647_2170-5490	gi|83288218|sp|Q53GS7.2|GLE1_HUMAN	3.00E-36	Nucleoporin GLE1	mRNA export
					scaffold-76647_6723-7102	gi|464865|sp|Q04202.1|TCB2_CAEBR	6.00E-20	Transposable element Tcb2 transposase	Transposable element
78628*	78598	5211	5144	126	scaffold-78598_2145-2291	gi|9910620|sp|O43715.1|TRIA1_HUMAN	5.00E-26	TP53-regulated inhibitor of apoptosis 1	Apoptosis
78917	183364	2847	2914	102	scaffold-183364_3863-4414	gi|464864|sp|P35072.1|TCB1_CAEBR	9.00E-36	Transposable element Tcb1 transposase	Transposable element
80526	232830	3500	3567	126	scaffold-232830_2720-3610	gi|74821373|sp|Q95SX7.1|RTBS_DROME	3.00E-17	Probable RNA-directed DNA polymerase from transposon BS	RNA-dependant DNA replication
88186	336919	2690	2757	126	scaffold-336919_2507-2698	gi|81898999|sp|Q8C627.1|F221B_MOUSE	5.00E-16	Protein FAM221B	*Unknown*
93783	334686	526	459	126	scaffold-334686_207-1019	gi|134035372|sp|Q6A085.2|ZN629_MOUSE	1.00E-30	Zinc finger protein 629	Transcriptional regulation
97205	34582	2640	2706	124	scaffold-34582_9401-10219	gi|464864|sp|P35072.1|TCB1_CAEBR	3.00E-37	Transposable element Tcb1 transposase	Transposable element
					scaffold-34582_17105-17316	gi|50403735|sp|P33005.2|KALM_CHICK	2.00E-29	Anosmin-1	Cell adhesion
101670*	183364	2838	2766	97.1	scaffold-183364_3863-4414	gi|464864|sp|P35072.1|TCB1_CAEBR	9.00E-36	Transposable element Tcb1 transposase	Transposable element
110970*	12855	20623	20586	71.3	scaffold-12855_2228-14681	gi|338817941|sp|Q9QXZ0.2|MACF1_MOUSE	1.00E-62	Microtubule-actin cross-linking factor 1	*Multiple*
	32096	8942	9009	121	scaffold-32096_1541-5542	gi|338817989|sp|Q9UPN3.4|MACF1_HUMAN	2.00E-103	Microtubule-actin cross-linking factor 1, isoforms 1/2/3/5	*Multiple*
114899	84221	5438	5371	126	scaffold-78598_3908-8097	gi|145566945|sp|Q2NKQ1.2|SGSM1_HUMAN	3.00E-63	Small G protein signaling modulator 1	Golgi apparatus
					scaffold-84221_5333-6762	gi|82100806|sp|Q8AXC7.1|PGFRA_TAKRU	1.00E-92	Platelet-derived growth factor receptor alpha	Chemotaxis and development
114971	340942	1	61	113	scaffold-340942_931-1155	gi|123792664|sp|Q0V8T9.1|CTP5A_MOUSE	3.00E-21	Contactin-associated protein like 5-1	Cell adhesion
123874*	228433	1365	1432	121	scaffold-228433_10-3697	gi|425906075|sp|Q8NF91.4|SYNE1_HUMAN	2.00E-22	Nesprin-1	Cytoskeleton and organelle
131325	300418	2369	2302	126	scaffold-300418_521-4385	gi|17367145|sp|Q9ULA0.1|DNPEP_HUMAN	6.00E-19	Aspartyl aminopeptidase	Protein metabolism
136924	64513	1788	1721	126	scaffold-64513_1202-1897	gi|75056157|sp|Q9GLY5.1|ITIH3_RABIT	6.00E-37	Inter-alpha-trypsin inhibitor heavy chain H3	Hyaluronan metabolism

	272191	699	766	121	scaffold-272191_683-862	gi|3024056|sp|Q63416.1|ITIH3_RAT	9.00E-17	Inter-alpha-trypsin inhibitor heavy chain H3	Hyaluronan metabolism
138429	232830	3491	3424	99	scaffold-232830_2720-3610	gi|74821373|sp|Q95SX7.1|RTBS_DROME	3.00E-17	Probable RNA-directed DNA polymerase from transposon BS	RNA-dependant DNA replication
147541*	392290	308	375	126	scaffold-392290_2121-2231	gi|75077434|sp|Q4U0T9.1|CSRP3_BOVIN	4.00E-18	Cysteine and glycine-rich protein 3	Cardiac muscle development
155423	31593	2825	2892	121	scaffold-31593_6120-6725	gi|464864|sp|P35072.1|TCB1_CAEBR	6.00E-36	Transposable element Tcb1 transposase	Transposable element

* Annotated SNP with potential divergent selection.
