# Supplementary material for: RAD-QTL Mapping Reveals Both Genome-Level Parallelism and Different Genetic Architecture Underlying the Evolution of Body Shape in Lake Whitefish (Coregonus clupeaformis) Species Pairs
Source: G3 (Bethesda). 2015 May 21;5(7):1481–91. doi: 10.1534/g3.115.019067 (PMC4502382; doi:10.1534/g3.115.019067)
Supplement: Blog post: Multiple Paths to the Same Result: Parallel Evolution in Lake Whitefish [file supp_5_7_1481_v2_index.html]

Blog post: Multiple Paths to the Same Result: Parallel Evolution in Lake Whitefish 

# RAD-QTL Mapping Reveals Both Genome-Level Parallelism and Different Genetic Architecture Underlying the Evolution of Body Shape in Lake Whitefish (*Coregonus clupeaformis*) Species Pairs

## Multiple Paths to the Same Result: Parallel Evolution in Lake Whitefish

For Lake Whitefish, history has repeated itself. Across the St. John River region that spans Qu?bec and Maine, these freshwater fish have continually evolved in the same way. Within the many individual lakes in this area, Lake Whitefish have diverged into two groups differentiated by size and body shape. These two groups, known as 'dwarf' and 'normal, give geneticists a powerful model to study parallel evolution. Read more at Genes to Genomes
