# Supplementary material for: RAD-QTL Mapping Reveals Both Genome-Level Parallelism and Different Genetic Architecture Underlying the Evolution of Body Shape in Lake Whitefish (Coregonus clupeaformis) Species Pairs
Source: G3 (Bethesda). 2015 May 21;5(7):1481–91. doi: 10.1534/g3.115.019067 (PMC4502382; doi:10.1534/g3.115.019067)
Supplement: Corrigendum [file supp_5_7_1481_v3_index.html]

Corrigendum 

# RAD-QTL Mapping Reveals Both Genome-Level Parallelism and Different Genetic Architecture Underlying the Evolution of Body Shape in Lake Whitefish (*Coregonus clupeaformis*) Species Pairs

## Corrigendum for Laporte *et al*., 2015

**Files in this Data Supplement:**

- Corrigendum - Corrigendum for Laporte *et al*., 2015.
