# Supplementary material for: RAD-QTL Mapping Reveals Both Genome-Level Parallelism and Different Genetic Architecture Underlying the Evolution of Body Shape in Lake Whitefish (Coregonus clupeaformis) Species Pairs
Source: G3 (Bethesda). 2015 May 21;5(7):1481–91. doi: 10.1534/g3.115.019067 (PMC4502382; doi:10.1534/g3.115.019067)
Supplement: Corrigendum [file supp_g3.115.019067_Laporte_corrigendum.pdf]

Corrigendum for Laporte *et al.*, *G3 5* (7) 1481-1491.

G3: *Genes* | *Genomes* | *Genetics* Vol 5, 1481-1491, July 2015, Copyright © Laporte *et al*.

CORRIGENDUM

In the article by M. Laporte, S. M. Rogers, A.-M. Dion-Côté, E. Normandeau, P.-A. Gagnaire, *et al*. (*G3 5*: 1481-1491) entitled "RAD-QTL Mapping Reveals Both Genome-Level Parallelism and Different Genetic Architecture Underlying the Evolution of Body Shape in Lake Whitefish *(Coregonus clupeaformis)* Species Pairs" on pages 1484 and 1485, the legends for Figures 2 and 3 were erroneously reversed. As a result of this error, the legends for these figures are incorrect and should be exchanged, as follows:

Figure 2. Fish shape variation among individuals along PC1 and PC2 in all five lakes. Each dot represents an individual (black color = normal species; white color = dwarf species). The means fish shape for each species lakes combination were also indicated (circle = Cliff; square = East; diamond = Indian; upper triangle = Témiscouata and lower triangle = Webster).

Figure 3. Mean shape comparisons between normal (white dot; dashed line) and dwarf Whitefish (black dot; full line) for Cliff, East, Indian, Témiscouata, and Webster lakes. Red arrows show how shape changes from normal to dwarf Whitefish.
